# Limited benefit of repeating a sensitive question in a cross-sectional sexual health study

**DOI:** 10.1186/1471-2288-13-34

**Published:** 2013-03-09

**Authors:** Abigail Norris Turner, Prabasaj Paul, Alison H Norris

**Affiliations:** 1 Division of Infectious Diseases, College of Medicine, Ohio State University, Columbus, OH, USA; 2 Division of Epidemiology, College of Public Health, Ohio State University, Columbus, OH, USA

**Keywords:** Sensitive behavior, ACASI, Sexually transmitted disease, Tanzania

## Abstract

**Background:**

Sexual health research relies heavily on self-reported data. We explored whether repeating a key measure – number of lifetime sexual partners – improved the validity of this self-reported response.

**Methods:**

Using data from a study of Tanzanian plantation residents, we examined which of 505 participants changed their responses when a question about sexual partners was repeated. We examined which variable (first, second, or maximum response) was more predictive of herpes simplex virus type 2 (HSV-2) seropositivity, a biomarker strongly associated with number of lifetime partners. HSV-2 status was assessed using the HerpeSelect 2 ELISA IgG test.

**Results:**

When asked a second time, 10.7% of participants increased and 3.6% decreased their reported number of partners. Participants using audio computer-assisted self-interviews were more likely to change than those interviewed in person (p = 0.006). The increased odds of HSV-2 seropositivity with each additional partner ranged from 10% to 13% in men, and 33% to 37% in women, depending on which partner variable was used. Estimates had considerable confidence interval overlap and no substantial differences in precision.

**Conclusions:**

Some participants change their responses when asked a sensitive question a second time, but in this population, changes did not meaningfully affect associations between lifetime partners and HSV-2.

## Background

Sexual health research is heavily reliant on self-reported behavioral data. Although the limitations of self-reported data are widely accepted [1], alternatives are often unavailable. Audio computer-assisted self-interviewing (ACASI) has been shown in some cases to improve validity of self-reported data [2,3]. Staff training and gender-matched interviewing may improve self-reported data, but limited research has investigated these effects [4]. Prostate specific antigen (PSA) has been used in some studies to validate self-reports of unprotected vaginal sex in women [5], but PSA measurement is appropriate only for recent sex. Many sensitive behaviors for which there are no biomarkers are routinely measured for sexual health research, including the number of lifetime sex partners.

We explored whether simply repeating a question about participants’ number of lifetime sexual partners could be an inexpensive, simple method to improve the validity of self-reported responses to this question. A cross-sectional survey was administered to male and female agricultural plantation residents in Tanzania [6]. We examined (a) whether participants changed their responses to this question when asked a second time; (b) which participant characteristics were associated with changing responses; and (c) whether asking the same question twice makes for a “better” screening test for herpes simplex virus type 2 (HSV-2) seropositivity.

## Methods

### Parent study

Across Africa, millions of men and women working on agricultural plantations represent a unique, understudied, and potentially vulnerable population. The parent study was conducted at one such plantation located in northern Tanzania, which employs about 3,800 people. One author (AHN) conducted an observational study in 2004 to assess how the specific context of life on an agricultural plantation influences sexual behavior and risks for several sexually transmitted infections (STIs), including HIV, syphilis and HSV-2 [6]. The parent study enrolled agricultural plantation residents aged 18 years or older, who were able to give consent, and were randomly selected or volunteered in person at the study site [6]. The team used a mobile research unit to administer a questionnaire and conduct STI/HIV testing. A total of 556 participants completed detailed questionaires; of these, 505 had complete data on number of sexual partners; 513 provided biological specimens for HSV-2 testing; and 410 had both complete questionnaire and HSV-2 data.

The survey was administered in Swahili. Many sensitive topics were addressed, and thus ACASI was utilized in an attempt to increase honesty in participant responses. Each participant heard the questions in a gender-matched voice. Most participants (82.0%) completed the ACASI survey independently, although individuals with limited literacy or inability to use the computer (16.0%) were assisted by a gender-matched interviewer who entered participants’ reponses on the computer. Mode of administration was missing for 2.0%; these respondents are excluded from multivariate models.

Correct information on participants’ lifetime number of sexual partners was thought to be of central importance to the study. Thus, in addition to the efforts described above to increase honest self-report, we asked about lifetime number of sexual partners two times, with the second question following immediately after the first question, prefaced with an explanation about the importance of the participants’ honest response. The survey included the following two questions, modified so women were asked about male partners and men about female partners:

Q1 Please, can you tell me how many women/men you have had sex with in your life? Include your current partner and all sexual partners you have had in your life, even if you were forced. (If you aren’t sure, please estimate).

Q2 Because this question is very important, I am going to ask you again. Please tell me honestly, without hiding anything, to the best of your ability. In your life, how many women/men have you had sex with, whether you agreed or you were forced? Include the person you had sex with first, your current partner, and all other sexual partners from your whole life.

Respondents who answered Q1 and then selected “not applicable” for Q2 (n = 78) were assumed to have already provided their most valid response to Q1, thus the analyzed value of Q2 for these individuals was the same as Q1. Number of partners was coded as an ordinal categorical variable, with values 0–9 corresponding to the reported number of partners, 10 corresponding to 10–14 partners, 11 corresponding to 15–19 partners, and 12 corresponding to ≥20 partners. (Categories 10, 11 and 12 were assigned to divergent groups based on the frequencies of the raw data, justified by relative sparsity in the higher categories).

HSV-2 testing was conducted on serum using HerpeSelect 2 ELISA IgG test (Focus Technologies, Cypress, CA).

### Ethical approval

The parent study was approved by the Tanganyika Planting Company Ethical Committee, Kilimanjaro Christian Medical College Ethics Committee, Tanzanian National Institute of Medical Research, Tanzanian Commission on Science and Technology, and Yale University’s Human Investigations Committee. The Ohio State University Institutional Review Board determined that these secondary analyses were exempt from further review.

### Statistical analysis

Data analysis was performed using R (2.10.0 through 2.14.0). We used frequency statistics to evaluate whether respondents changed responses and in which direction (increased or decreased), and whether any variable (sex, age, mode of survey administration, HSV-2 status) was associated with changing responses. We used Fisher’s exact test because of small expected cell sizes. Statistical signficance was set at α = 0.05.

We used separate multivariable logistic regression models to examine the association between self-reported number of partners and HSV-2: Model A used Q1 as the independent variable, Model B used Q2, and Model C used the maximum of Q1 and Q2. Number of partners satisfied the assumption of linearity in the log odds of HSV-2 positivity. Because of biologically plausible and statistically meaningful (based on Akaike information criterion, see below) differences between men and women, for each model we included a product-interaction term between sex and number of partners. We first computed unadjusted odds ratios (ORs) and 95% confidence intervals (CI) for the effect of increasing lifetime partners on HSV-2 seropositivity, seperately for men and women. We then computed estimates adjusting for respondent age and mode of survey administration. Precision was assessed using the confidence limit ratio (CLR: ratio of upper to lower bounds of the 95% CI). Models were compared using the Akaike information criterion (AIC) and area under the receiver operating characterictic curve (AUC).

## Results

### Changes in responses

Among 505 individuals (250 men and 255 women) with valid sexual partner data, 85.7% did not change their reported number of lifetime sex partners when questioned a second time (Table 1). Among those who did change, 10.7% increased and 3.6% decreased the number of partners. Only mode of survey administration was associated with changing responses (*p* = 0.006, Table 1). Participants using ACASI were significantly more likely to change their responses than those assisted by an interviewer (n = 81, 16.0% of the study population). HSV-2-seropositivity was marginally associated with changing answers (*p* = 0.073, Table 1).

**Table 1:** Associations between participant characteristics and changing responses to the number of lifetime sexual partners

	**No change**		**Increase**		**Decrease**		*p*-value*
	**n**	**(%)**	**n**	**(%)**	**n**	**(%)**	
**Total**	433	(85.7%)	54	(10.7%)	18	(3.6%)	
**Sex**							
**Male**	209	(83.6%)	28	(11.2%)	13	(5.2%)	0.124
**Female**	224	(87.8%)	26	(10.2%)	5	(2.0%)	
**Mode**							
**Self-administered**	345	(83.5%)	50	(12.1%)	18	(4.4%)	0.006
**RA-administered**	78	(96.3%)	3	(3.7%)	0	(0.0%)	
**Missing**	11	(90.9%)	1	(9.1%)	0	(0.0%)	
**HSV-2 status**							
**HSV-2 negative**	158	(88.8%)	14	(7.9%)	6	(3.4%)	0.073
**HSV-2 positive**	188	(81.0%)	35	(15.1%)	9	(3.9%)	
**Missing**	87	(91.6%)	5	(5.3%)	3	(3.2%)	
**Age (years)**							
**14-21**	38	(77.6%)	7	(14.3%)	4	(8.2%)	0.428
**22-29**	134	(87.6%)	12	(7.8%)	7	(4.6%)	
**30-37**	135	(87.1%)	18	(11.6%)	2	(1.3%)	
**38-45**	74	(85.1%)	10	(11.5%)	3	(3.4%)	
**46+**	40	(83.3%)	6	(12.5%)	2	(4.2%)	
**Missing**	12	(92.3%)	1	(7.7%)	0	(0.0%)	

* p-values computed using Fisher’s exact test, with missing values excluded.

### Number of partners and HSV-2

**Figure 1:**
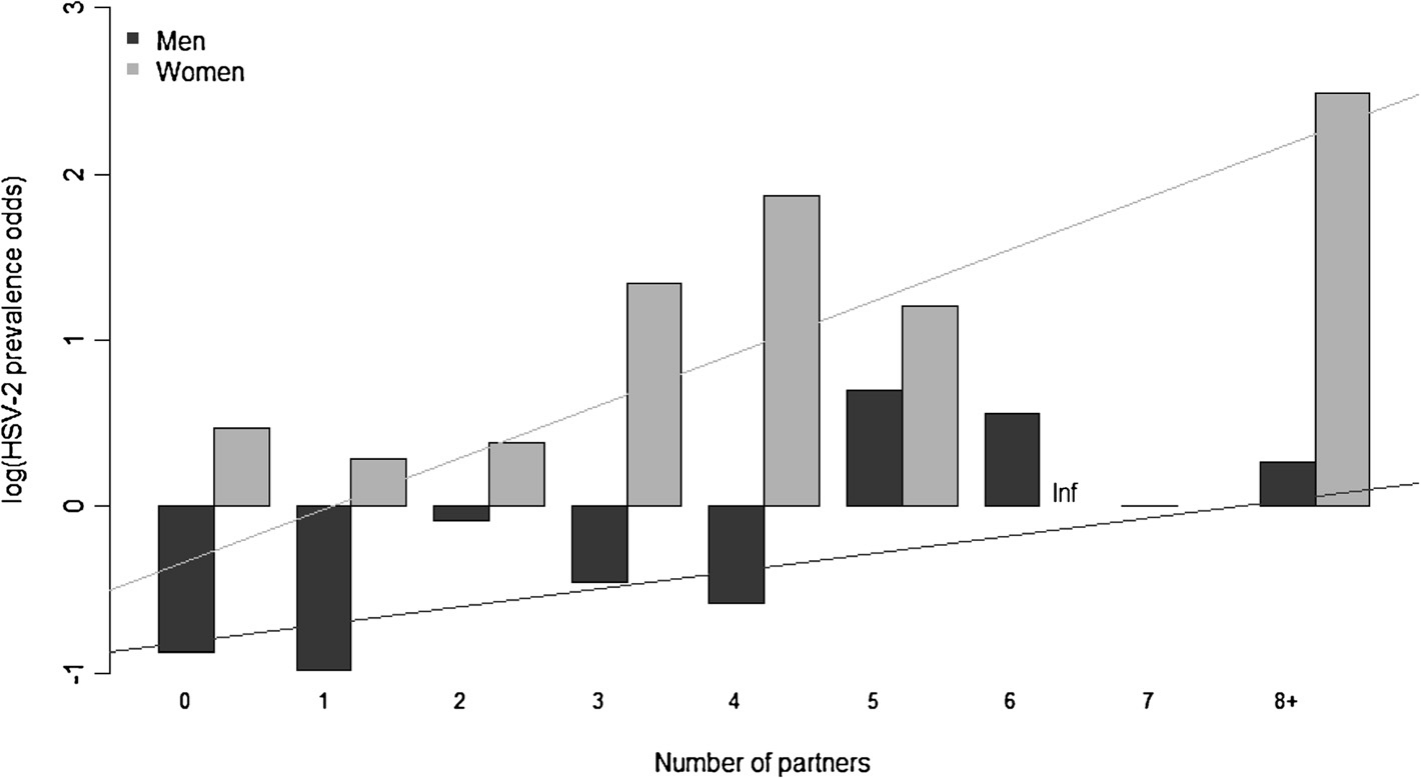
Log odds of prevalent HSV-2 infection according to number of partners, with partners measured as the larger of Q1 and Q2.

Among the 410 individuals with both valid questionnaire and HSV-2 data, the odds of HSV-2 seropositivity increased with increasing lifetime sexual partners (Figure 1). Because the differences between men and women were determined to be statistically meaningful based on AIC, we examined associations seperately for men and women in all subsequent analyses.

**Figure 2:**
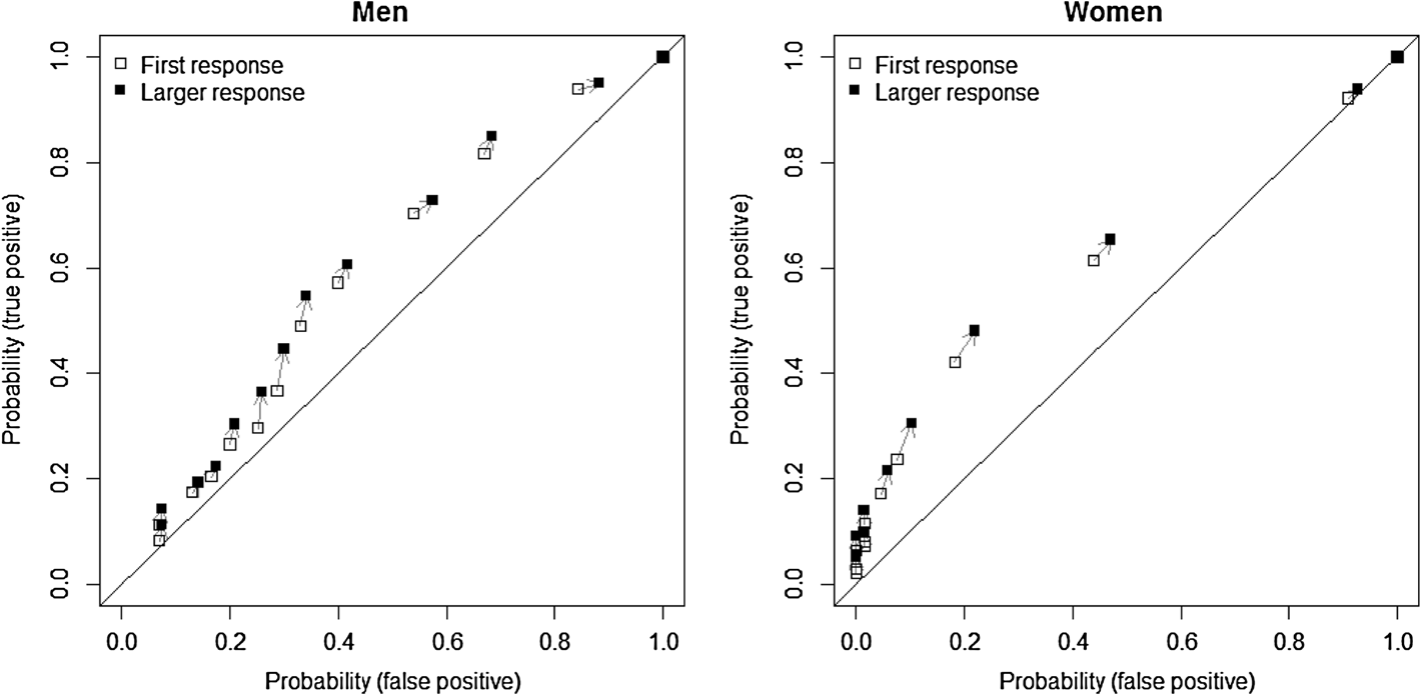
Changes in receiver operating characteristic (ROC) curves between using Q1 as the classifier and using the larger of Q1 and Q2 as the classifier.

We examined ROC curves showing various cut-points for binary classification of HSV-2 positivity, using the response to Q1 alone and the response using the larger of Q1 and Q2, seperately by sex (Figure 2). Using the larger of the two responses as the classifier netted as many or more cases (true positives) as well as non-cases (false positives), shifting the ROC curve overall upward and to the right and thus marginally increasing the area under the curve.

### Multivariable modeling

When Q1 was the independent variable (Model A), men had an unadjusted 10% increase in odds of HSV-2 infection per one-partner increase. Women’s corresponding one-partner increase using Q1 was 33% (Table 2). When Q2 was the independent variable (Model B), the unadjusted increase in odds of HSV-2 positivity were slightly higher: 13% and 35% for men and women, respectively. In Model C the larger of the responses to Q1 and Q2 was the independent variable. Model C produced similar results to Model B, with unadjusted increases in odds of HSV-2 positivity of 13% for men and 37% for women, per one-partner increase. For all models, adjustment for age and interview mode did not meaningfully change estimates (Table 2). Across all models, the estimates for men differed considerably from women, but within each sex we observed extensive overlap in CIs in all estimates.

**Table 2:** Unadjusted and adjusted logisitic regression models using the first-reported lifetime number of partners (Q1), the second-reported lifetime number of partners (Q2), and the maximum of the first and second responses and odds of HSV-2 seropositivity

	Model A*				Model B*				Model C*			
	**Unadjusted**		Adjusted **		**Unadjusted**		Adjusted **		**Unadjusted**		Adjusted **	
	**OR**	**(95% CI)**	**OR**	**(95% CI)**	**OR**	**(95% CI)**	**OR**	**(95% CI)**	**OR**	**(95% CI)**	**OR**	**(95% CI)**
**Men**	1.10	(1.02, 1.19)	1.08	(1.00, 1.18)	1.13	(1.05, 1.22)	1.11	(1.02, 1.21)	1.13	(1.04, 1.22)	1.11	(1.02, 1.21)
**Women**	1.33	(1.20, 1.47)	1.30	(1.17, 1.44)	1.35	(1.22, 1.49)	1.33	(1.20, 1.47)	1.37	(1.24, 1.51)	1.35	(1.22, 1.49)
**CLR**	1.17 (M); 1.22 (W)		1.18 (M); 1.23 (W)		1.17 (M); 1.22 (W)		1.18 (M); 1.22 (W)		1.17 (M); 1.22 (W)		1.18 (M); 1.22 (W)	
**AIC**	517.83		484.98		511.27		480.25		510.10		478.65	
**AUC**	0.66		0.74		0.67		0.74		0.67		0.75	

Abbreviations**:** OR: Odds ratio; CI: confidence interval; HSV-2: herpes simplex virus type 2; CLR: confidence limit ratio; AIC: Akaike information criterion; AUC: area under the receiver operating characterictic curve; M: men; W: women.

* Model A uses Q1 as the independent variable; Model B uses Q2 as the independent variable; Model C uses the maximum of Q1 and Q2 as the independent variable. All models are based on records with no missing value for Q1, Q2, sex, age, mode of survey administration and HSV-2 seropositivity.

** Adjusted for age and mode of survey administration.

Considering men and women seperately, precision was approximately equal across all models (Table 2). AUC was similar across all unadjusted models, and also across all adjusted models. The AIC decrease from Model A to Model B indicates improved model fit using Q2 over Q1. The larger AUC in the adjusted models indicates that these perform better than the unadjusted models.

## Discussion

This simple analysis examined whether repeating a question about a sensitive self-reported behavior led to significant changes in responses. To our knowledge, no other study has evaluated the benefit of repeating a sensitive question. Approximately 1 out of 7 participants changed their responses when asked a second time; as expected, considerably more respondents increased rather than decreased their self-reported number of partners. However, using changed responses in multivariable models did not significantly strengthen associations between lifetime number of partners and odds of HSV-2 seropositivity, nor improve precision of those estimates. The only improvement obtained by asking the question a second time was in model fit.

The only significant predictor of changing response was interview mode: participants using ACASI independently were considerably more likely to revise their responses. Although existing research about sensitive topics as measured by ACASI is somewhat mixed, the majority indicates increased endorsement of sensitive behaviors when ACASI is used [7]. Our findings confirm that participants were more comforable revising their responses with the more private computer interface.

Our analysis has a number of limitations. As in other studies measuring sensitive behavior, we assumed that a higher reported number of sexual partners was more valid. Our question also specified heterosexual partnerships, which represent the majority but not the entirety of sexual partnerships on the plantation. We detected significant differences in participants’ willingness to change their responses by interview mode (ACASI vs. interviewer-assisted), but only a small number of individuals completed the survey with the help of an interviewer. Finally, we chose HSV-2 seropositivity as a proxy biomarker of lifetime sexual partners, given the extensive literature documenting this association. Whether our findings about the value of asking a question a second time would hold for other behaviors or outcomes is unknown.

## Conclusion

In summary, repeating a question about lifetime sexual partners showed that some participants do change their responses, but that the change does not meaningfully affect the observed associations between lifetime partners and HSV-2 risk.

## Competing interests

The authors declare that they have no competing interests.

## Authors’ contributions

ANT designed the analysis and contributed extensively to drafting and revising the manuscript. PP carried out the analysis and wrote the first draft of the manuscript. AHN designed and collected the data for the parent study and revised the manuscript. All authors (ANT, PP, AHN) read and approved the final manuscript.

## Pre-publication history

The pre-publication history for this paper can be accessed here:

http://www.biomedcentral.com/1471-2288/13/34/prepub
